# Understanding the Shape-Memory Alloys Used in Orthodontics

**DOI:** 10.5402/2011/132408

**Published:** 2011-10-03

**Authors:** Daniel J. Fernandes, Rafael V. Peres, Alvaro M. Mendes, Carlos N. Elias

**Affiliations:** ^1^Department of Orthodontics, State University of Rio de Janeiro, Rio de Janeiro, RJ, Brazil; ^2^Biomaterials Laboratory, Military Institute of Engineering, Rio de Janeiro, RJ, Brazil

## Abstract

Nickel-titanium (NiTi) shape-memory alloys (SMAs) have been used in the manufacture of orthodontic wires due to their shape memory properties, super-elasticity, high ductility, and resistance to corrosion. SMAs have greater strength and lower modulus of elasticity when compared with stainless steel alloys. The pseudoelastic behavior of NiTi wires means that on unloading they return to their original shape by delivering light continuous forces over a wider range of deformation which is claimed to allow dental displacements. The aim of this paper is to discuss the physical, metallurgical, and mechanical properties of NiTi used in Orthodontics in order to analyze the shape memory properties, super-elasticity, and thermomechanical characteristics of SMA.

## 1. Introduction

Nickel-titanium-based shape memory alloys have been widely used in orthodontics due to their good mechanical properties, biocompatibility [1], ductility, resistance to corrosion [2], lower elastic modulus, and special characteristics such as superelasticity and shape memory effect [3].

**Figure 1:**
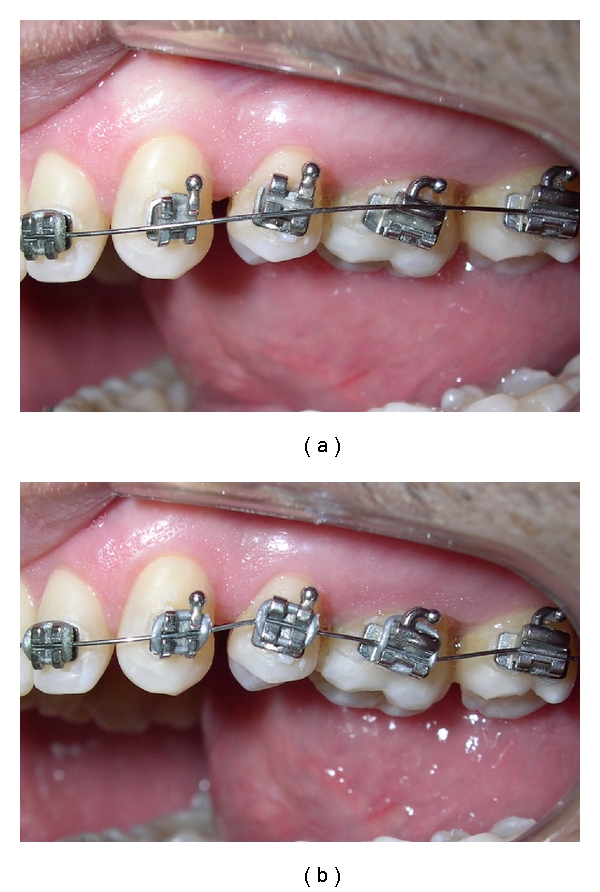
Maxillary arch during initial alignment orthodontic stage. Superelastic NiTi 0.012-inch (a) before and (b) after bracket engagement. Note the degree of the misalignment which the wire can tolerate due to its superelastic properties.

The superelasticity means that on unloading, the wire may return to its original shape before loading. The alloy can be deformed until 7-8% strain, and it represents almost forty-times the stainless steel capacity. During loading (Figure 1), periodontal-ligament is deformed and deactivation starts upon a plateau of light forces appreciated to provide the optimum biological tooth movement while the crystalline structure of the alloy returns to its original arrangement [4–6].

Shape-memory alloys are materials that can remember their original shape, after being elastically or pseudoplastically deformed by increasing their temperature. The memory effect is due to thermoelastic martensitic transformation which can be described as a first-order displacive nondiffusional process [7]. Herein a body-centered cubic parent phase (austenitic phase) transforms by a shear mechanism into a orthorhombic or monoclinic martensite phase. This process can pass or not through an intermediate tetragonal phase (R phase) [7–9]. 

The objective of this overview is to analyze the physical metallurgical and mechanical properties of NiTi used in orthodontics, understand the thermomechanical behavior and summarize the phase transformation characteristics in order to analyze the shape memory effect and superelasticity of the SMA.

## 2. Crystal Structure of Nickel-Titanium Alloy

All alloys with SME show a change in their lattice structure or atomic arrangement, characterizing a phase change while receiving or releasing thermal energy.

The critical deformation and shape recovery are explained according to the lattice parameters changes through a phase transformation between austenite and martensite and the characteristics of the crystal structure [10]. 

The lattice parameters of NiTi alloy at high temperature ranges is a stable, body-centered cubic structure which is referred to as the austenite or parent phase. NiTi alloys have the particular characteristic that when they are cooled through a critical-transformation temperature range, the alloy shows changes in its modulus of elasticity (stiffness), yield strength, and electric resistivity as a result of changes in interatomic bonding. By reducing or cooling the temperature through this range, there is a change in the crystal structure which is known as the martensitic transformation; the amount of this transformation is a function of the start (Ms) and finish (Mf) temperature (Figure 5). The event causes alterations in the physical properties of the alloy [11] and allows features of shape memory [12]. 

## 3. Martensitic Transformation

**Figure 2:**
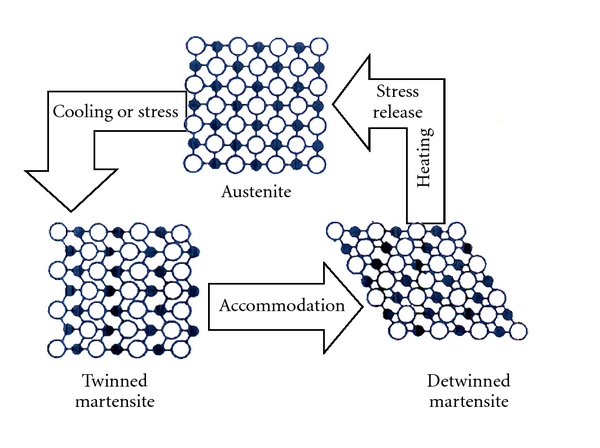
Changes in crystal form of SMA which leads to Superlasticity (induced by stress) and SME (induced by heating). Adapted from [24].

**Figure 3:**
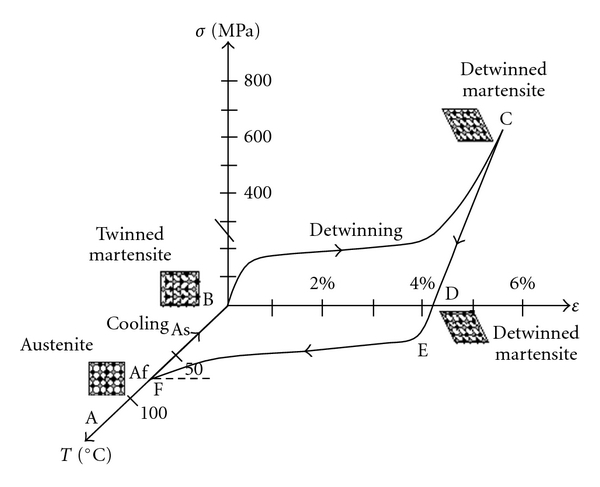
Stress-strain-temperature exhibiting the shape memory effect for NiTi alloy. The crystal changes are also related to temperature and stress. Adapted from [39].

The martensitic transformation is an instantaneous thermoelastic first-order crystalline displacive military process, with diffusionless characteristics in solids, in which atoms move cooperatively, and often by a shear-like mechanism. During transformation, a body-centered cubic parent phase (austenite) shear and gives rise to twinned martensite (Figures 2 and 3) that forms the structure of a closely packed hexagonal lattice with an orthorombic or monoclinic arrangement [7]. This change in solid state allows properties as single (one-way), double (two-way) shape memory effect and superelasticity [7–9].

## 4. Shape Memory Effect

The shape memory effect allows the alloy to return to its previous shape, recovering from large strains through heating. The formation of directional and strong interatomic bonds are responsible to pull back the displaced structures to their previous positions.

All alloys with SME show a change in their lattice structure or atomic arrangement, characterizing a phase change while receiving or releasing thermal energy. This change in NiTi lattice structure is from a high temperature ordered body-centered cubic austenite form (B2 form or parent phase) to a low-temperature orthorombic face-centered cubic shape (B19 form) or a monoclinic face-centered cubic shape (B19′ form). Monoclinic phase has no equal sides in the crystal cell and no right angles as it was tilted or squashed. An intermediate trigonal phase between austenite and martensite called R phase can occur as a result of a rhombohedral distortion of the cubic parent phase. Thin plates of R phase nucleate from dislocations (B2 to R), grow and join together while many other plates forms until the entire region changes into R phase [13]. When the plates shrink and disappear, the phase is reverted to austenite. The reversibility of the process can be induced by heating the alloy above the transformation temperature (Figures 2 and 3). The alloy structure returns to its original austenite body-centred cubic parent phase, which is more stable at high-temperature conditions [12].

## 5. Superelastic Effect

**Figure 4:**
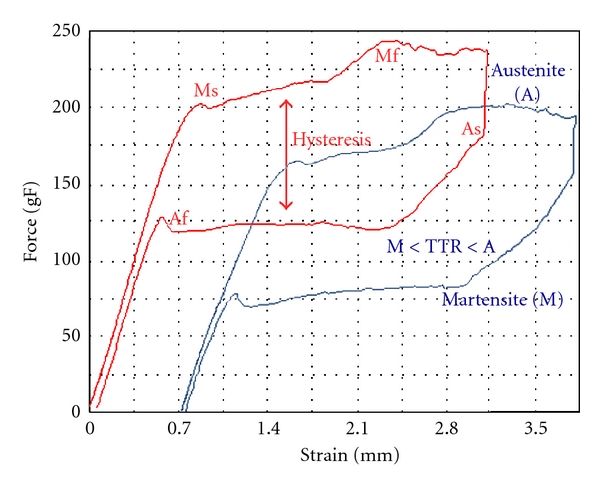
Supereleastic (red) and thermal activated (blue) NiTi 0.012-inch orthodontic archwires during three-point bending test. Note different yielding points (*Y*1, *Y*2), Martensite and Austenite start (Ms, As) and finish (Mf, Af) phases transformation and the temperature of transformation (TTR).

Besides the thermal energy, stress can also induce the transformation from austenitic to martensitic phase. When sufficient shear stress is applied to an austenitic alloy, martensitic transformation starts in a way to relief the excessive stress applied (Figure 2). While stress is maintained, the material stays in martensitic phase and remains deformed. When shear is removed, the stress remains in a level where the martensite is not stable and the phase is reverted to austenite. The intense deformation capacity of the alloy (up to 7-8% strain) and the reverse deformation at lower stress are called superelasticity or pseudoelasticity (Figure 4). 

Pseudoplastic behavior is assigned to situations when the plastic deformation remains recoverable without entering the plastic stage. The amount of plastic deformation that occurs in NiTi alloys is recoverable within certain limits. An irreversible process can be established after excessive stress is maintained, inducing increase of dislocation density which restricts growth of martensite phase and development of stress-induced reoriented martensite resulting in increased transformation hardening [14–17].

## 6. Stress-Strain Curve

Figure 4 is a stress-strain curve of 0.014-inch orthodontic superelastic (in red color) and thermal-activated (in blue color) archwire, probably of an Ni-50Ti alloy (at %). Under low loading, the first yielding occurs (*Y*1) after initial loading, which is the starting point of deformation due to rearrangement of the R phase variants. This first step gives rise to an strain of 0.8%.

The second yielding point (*Y*2) occurs at the starting point of the deformation due to stress-induction of the B19′ martensite from the R phase. This stage gives rise elongation up to 7% including that of the R phase. The rate of stress strain becomes larger and a loading plateau begins.

 The subsequent elastic recovery in the unloading stage is due to B2 transformation after stress release where the martensite phase becomes unstable. The unloading plateau is lower than the load one and therefore a hysteresis loop is formed. Superelasticity shape recovery takes place after unloading, and no permanent strain was observed. For shape memory effect, the alloy might be warmed and the residual strain evaluated after the temperature above Af was reached. 

## 7. Hysteresis

Hysteresis could be generally described as an effort to return to a previous situation, concerning SMA it refers to the previous shape before deformation. The process results from phase transitions that is caused by the internal friction generated by the movement of the austenite-martensite interface (Figure 4). The friction force results from the movement in interatomic range and gives arise to energy dissipation which also increases the frictional energy against interfacial movement during phase transformation [18]. This energy dissipation due to lattice defects (dislocations) is irreversible and contributes to the transformation hysteresis [17, 19].

Martensite twins arises from the intent to relieve stress and accommodate the shape, volume, and lattice incompatibility between austenite and martensite. Accordingly, the process is caused by the irrecoverable dissipated energy due to formation of martensite twins and other lattice defects. Some elastic energy is dissipated in strain during phase transformation whereas the elastic energy stored during forward transformation is entirely released upon reverse transformation acting as driving and thus does not contribute to the transformation hysteresis [17, 19]. The hysteresis transformation could not have been expected when all lattice distortions were accommodated elastically and no lattice defects were formed.

Other factors which affect the hysteresis transformation are alloy composition including a third element insertion as Cu [20–22] and precipitates formation [23]. A large atomic size of the additional alloying elements may result in more frictional force thus more energy dissipation leading to increased hysteresis [18]. 

## 8. Transformation Mechanism

SME and superelasticity are associated with thermoelastic and stress-induced martensitic and its reverse phase transformation. The transformation temperature is related to the presence of lattice defects which are present since the solidification of the alloy during its production or can be induced during plastic deformation or by thermal stresses resulting from rapid cooling [14]. The number of dislocations and principally its density, may also be introduced when the alloy is stressed, although the dislocations slippage is a nondamaging process to the crystal structure in SMA. Meanwhile, the strain energy retained internally which would be associated with dislocations and plastic deformation in general metallic alloys, in SMA is stored and allows the process lead back to austenite form from martensite phase [14]. 

Moreover to the slippage, SMA may also store the strain energy by the formation of mechanical twins, or twinning (Figure 2). Twins result from a shear force which produces atomic displacements on twin boundary and accommodate the volumetric change associated with the transition between phases. The twinning is important because it can allow new slip systems in orientations relative to stress axis. An increase in the number of operable slip systems and the intensity of strain energy that can be stored takes place, resulting in a nondamaging process to the crystal structure [14].

## 9. Thermal Treatment and Transformation Temperature

SME can be associated with thermoelastic martensitic transformation, and heat is the agent that allows the reversion to austenite phase and the recovery of previous shape. While superelasticity is induced by stress, shape memory is initiated when the alloy in martensitic stage is warmed and thus, the austenite becomes the more thermodynamically stable phase. This transformation is governed by a specific range temperature (TTR) associated with heating and cooling process (Figure 4). This range is determined by the atomic composition and the state of structure which has been imparted by working and heat treatment of the alloy [24, 25]. The decrease in martensite start point (Ms) occurs due to introduction of lattice defects during the repeated motion of the austenite-martensite interface. It can be a result of the alloy annealing and may be reverted by thermo-mechanical treatment or aging [26]. 

During thermomechanical treatment by means of cooling, the single-step transformation is replaced by a two-step process from austenite to the R phase and then to martensite. In this case, the reverse transformation on heating may be as a single or two-step transformation [27]. The R phase transformation is responsible for changes in hysteresis which is smaller during a two-step transformation [16]. This intermediate phase also affects the SME strain, and in temperatures slightly above Ms it is seen like only a minor softness in elastic component.

## 10. Thermomechanical Properties

The thermomechanical properties of SMA are affiliated with thermoelastic martensitic transformation and can be governed by temperature during a thermally induced martensite transformation or by stress applied, as a result of a stress-induced martensitic transformation. This phase transformation and its reversion are accompanied by the variation of physical and mechanical properties [16] like yield strain, hardness, Young's modulus, bend resistance, roughness, expansion, internal friction, lattice spacing, electrical resistance, thermal conductivity, heat capacity, magnetic properties, and latent heat transformation [16]. Furthermore, the NiTi alloy is more ductile in the martensitic phase than the austenite phase.

## 11. Physical Properties Evaluation

According to the physical properties [28] alterations that arise during phase transformation, various techniques have been applied to study the transformation behavior of different shape memory alloys. Among them, different scanning calorimetry (DSC) [26, 28–32], electrical resistivity (ER) measurements, magnetic susceptibility measurements, and thermomechanical analysis (TMA) [33] are mainly employed. DSC is more employed than ER to evaluate transformation characteristics because ER process requires a laborious preparation of the samples and does not provide a precise indication of each phase temperature marks during transformation. X-ray diffraction (XRD) can also be employed to evaluate the phase transformation during thermal cycles by determining the austenite peaks.

**Figure 5:**
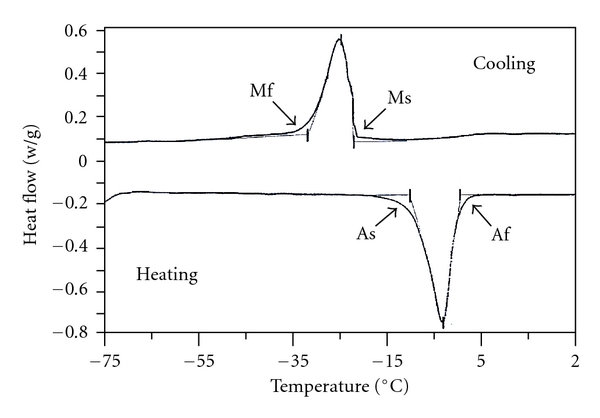
A schematic of a DSC curve for an SMA showing the phase transformations as peaks and the start and finish of martensite (Ms, Mf) and austenite (As, Af) phases during heating and cooling. Adapted from [24].

 The DSC testing requires very small samples, and a special test cell is able to slowly cool and heat the sample at a very precise rate. During cooling and heating, the cell indicates whether the sample is either giving off more heat or absorbing more heat in exothermic or endothermic reactions. The martensitic transformation is exothermic while the reverse transformation is considered endothermic and are seen in DSC as peaks or valleys in the temperature versus heat flow chart (Figure 5).

## 12. Other Factors Which Affect Mechanical Behavior, Phase Transformation and Microstructure

### 12.1. Grain Size

The reduction of grain size improves the pseudoelasticity and facilitates the orientation of neighbor grains and its stiffness is related to the number of martensite plates which are produced. These plates can be present in different orientations according to the interaction among the transformational strain with each new plate generated [34, 35]. The major cause of the pseudoelastic and critical strain supported by SMA is the transformational strain while the superelastic unloading recovery is a result of the disappearance of the martensite plates coupled with the reduction in transformational strain [35].

### 12.2. Alloy Composition

The composition of NiTi alloys induces the range of transition temperature and is responsible for variability in the number of electrons available for bonding. A very small excess nickel in structure can reduce TTR and increase the permanent yield strength of the austenite phase by roughly threefold. Moreover, the amount of nickel present in alloy may be controlled even during the ingot melting and casting process. Meanwhile, titanium-rich alloys contain a second phase Ti_2_Ni in the matrix and have higher transformation temperatures than those of the nickelless-rich or equiatomic NiTi alloys [36]. 

The transition temperature of a 50Ni-50Ti (at %) alloy is from −50 to +100°C. Reduction in TTR can be achieved during the manufacturing process due to changes in nickel-titanium ratio or by cold working and thermal treatment. NiTi alloy contains more nickel and when this approaches 60 at % Ni an alloy presents a lower shape memory effect though its ability to be heat-treated increases. Both 55- and 60-NiTi are more resilient, tougher, and have a lower modulus of elasticity than other alloys such as stainless steel, Ni-Cr, or Co-Cr [25].

### 12.3. Precipitates and Microstructure

The method of manufacturing, rolling, and intermediate annealing applied to NiTi wires during manufacturing process leads to breakage of the small crystal, and a recrystallization is necessary to develop a predictable average grain size and orientation. 

As a result of composition and thermal treatment, uniform grains may be formed or precipitates of some chemical constituents of the matrix may be acquired. These particles with disparate characteristics from the matrix, are constituents not thermodynamically stable under heat treatment temperature which can be combined with other constituents uniformly dispersed throughout the matrix. Ni_2_Ti and Ni_3_Ti precipitates allow the excess nickel to get off the crystal matrix, reduce the Ni content, and raise the TTR. Other precipitates as Ti_3_Ni_4_ may affect the mechanical behavior as strength of matrix austenite phase, improving the recoverability of the shape memory effect [20, 36]. 

### 12.4. Effect of Copper as a Ternary Alloying Addition Element

Copper- (Cu-) based SMA of contents from 5 to 15 at % of copper as a substituted for Ni, transforms in two steps under a sequence from cubic to orthorombic (B19) and then to monoclinic (B19′). With increase of Cu content, the transformation start temperature of the austenite to orthorombic martensite does not change while the second step from orthorombic to monoclinic decreases and only occurs in alloys of copper contents below 15 at % [3].

Changes in Cu-based alloys composition resulting from Cu addiction affect the mechanical behavior once hysteresis reduction, embrittlement, formability, and the Ms temperature and precipitations are also affected. The first step transformation hysteresis (B2 → B19) is much smaller than in binary alloys (B2 → B19′) due to reduction of interface movement during transformation [3, 37, 38]. Perhaps the rearrangement of martensite variants may be easier in Cu-NiTi alloys [13]. Additionally, Cu addition exceeding 10 at % embrittles the alloy and spoils the formability while the sensibility of Ms temperature is much reduced [13].

### 12.5. Effects of Oxygen and Carbon

The solid solubility of oxygen in NiTi alloy is very small and the addiction beyond this limit may contribute to increase of the contents of a primary phase of NiTi and an eutectic mixture of a solid solution of NiTi and oxides [18]. The oxygen combines with the nickel and titanium forming a complex oxide (Ti_4_Ni_2_O) which is present in NiTi as small oxide particles scattered throughout the structure [19]. The oxygen atoms consume two atoms of titanium for each atom of nickel twice as much Ti than Ni, resulting in the increasing of the nickel content in the remaining NiTi matrix, and they have an effect on the TTR [18]. The effects of nickel increase have been previously discussed.

Regarding some Ni-Ti-C ternary alloys, the carbon exists as carbides which show an increased hardness coupled with an inherent brittleness. TiC precipitates have the same environment effect seen from oxygen which ties up more titanium and provides alteration in the nickel balance with its increase [13].

## References

[1] Pandis N, Bourauel CP, Tomobe S (2010). Nickel-titanium (NiTi) arch wires: the clinical significance of super elasticity. *Seminars in Orthodontics*.

[2] Speck KM, Fraker AC (1980). Anodic polarization behavior of Ti-Ni and Ti-6A1-4V in simulated physiological solutions. *Journal of Dental Research*.

[3] Otsuka K, Wayman CM (1998). *Shape Memory Materials*.

[4] Lee JH, Park JB, Andreasen GF, Lakes RS (1988). Thermomechanical study of Ni-Ti alloys. *Journal of Biomedical Materials Research*.

[5] Serene TP, Adams JD, Saxena A (1995). *Nickel-Titanium Instruments: Applications in Endodontics*.

[6] Nikolai RJ (1997). Orthodontic wire: a continuing evolution. *Seminars in orthodontics*.

[7] Otsuka K, Wayman CM, Nakay K, Sakamoto H, Shimizu K (1976). Superelasticity effects and stress-induced martensitic transformations in CuAlNi alloys. *Acta Metallurgica*.

[8] Yang JH, Wayman CM (1992). Self-accomodation and shape memory mechanism of *ε*-martensite—I. Experimental observations. *Materials Characterization*.

[9] Yang JH, Wayman CM (1992). Self-accomodation and shape memory mechanism of *ε*-martensite—II. Experimental observations. *Materials Characterization*.

[10] Uehara T, Tamai T (2006). An atomistic study on shape-memory effect by shear deformation and phase transformation. *Mechanics of Advanced Materials and Structures*.

[11] Wang FE, Pickart SJ, Alperin HA (1972). Mechanism of the TiNi martensitic transformation and the crystal structures of TiNi-II and TiNi-III phases. *Journal of Applied Physics*.

[12] Thompson SA (2000). An overview of nickel-titanium alloys used in dentistry. *International Endodontic Journal*.

[13] Saburi T, Yoshida M, Nenno S (1984). Deformation behavior of shape memory TiNi alloy crystals. *Scripta Metallurgica*.

[14] Matsumoto H (1991). Effects of thermal cycling and annealing on the martensitic transformation of cold-rolled Ni_48_Ti_52_ alloy. *Materials Letters*.

[15] Filip P, Mazanec K (1995). Influence of work hardening and heat treatment on the substructure and deformation behaviour of TiNi shape memory alloys. *Scripta Metallurgica et Materiala*.

[16] Pattabi M, Ramakrishna K, Mahesh KK (2007). Effect of thermal cycling on the shape memory transformation behavior of NiTi alloy: powder X-ray diffraction study. *Materials Science and Engineering A*.

[17] Tong HC, Wayman CM (1975). Thermodynamics of thermoelastic martensitic transformations. *Acta Metallurgica*.

[18] Zarinejad M, Liu Y (2008). Dependence of transformation temperatures of NiTi-based shape-memory alloys on the number and concentration of valence electrons. *Advanced Functional Materials*.

[19] Olson GB, Cohen M (1975). Thermoelastic behavior in martensitic transformations. *Scripta Metallurgica*.

[20] Saburi T, Watanabe Y, Nenno S (1989). Morphological characteristics of the orthorhombic martensite in a shape memory Ti-Ni-Cu alloy. *ISIJ International*.

[21] Cui J, Chu YS, Famodu OO (2006). Combinatorial search of thermoelastic shape-memory alloys with extremely small hysteresis width. *Nature Materials*.

[22] Tadaki T, Okazaki H, Nakata Y, Shimizu KI (1990). Atomic configuration studied by ALCHEMI and X-ray diffraction of a stabilized M18R martensite in a *β* phase Cu-Au-Zn alloy. *Materials Transactions, JIM*.

[23] Tong YX, Liu Y, Xie ZL, Zarinejad M (2008). Effect of precipitation on the shape memory effect of Ti50Ni25Cu25 melt-spun ribbon. *Acta Materialia*.

[24] Hodgson DE, Brown JW (2000). *Using NiTi Alloys*.

[25] Callister WD (2008). *Materials Science and Engineering: An Introduction*.

[26] Miller DA, Lagoudas DC (2001). Influence of cold work and heat treatment on the shape memory effect and plastic development of NiTi. *Materials Science and Engineering A*.

[27] Miyasaki S, Igo Y, Otsuka K (1986). Effect of thermal cyclic deformation on the pseudoelasticity characteristics of Ti-Ni alloys. *Acta Metallurgica*.

[28] McCormick PG, Liu Y (1994). Thermodynamic analysis of the martensitic transformation in NiTi—II. Effect of transformation cycling. *Acta Metallurgica et Materialia*.

[29] Ma JL, Wu KH (2000). Short communication Effects of tantalum addition on transformation behaviour of (Ni51 Ti49 )1-x Tax and Ni50 Ti50-y Tay shape memory alloys. *Materials Science and Technology*.

[30] Matsumoto H (2003). Transformation behaviour with thermal cycling in NiTi alloys. *Journal of Alloys and Compounds*.

[31] Yinong L, Yong L, Humbeeck JV (1999). Two-way shape memory effect developed by martensite deformation in NiTi. *Acta Materialia*.

[32] Liu Y, Xie Z (2003). TEM in-situ study of the pre-strained NiTi shape memory alloy—driving force for shape recovery?. *Materials Science and Engineering*.

[33] Liu Y, Xie Z, Humbeeck JV (1999). Cyclic deformation of NiTi shape memory alloys. *Materials Science and Engineering A*.

[34] Pattabi M, Ramakrishna K, Mahesh KK (2009). Corrigendum to “Effect of thermal cycling on the shape memory transformation behavior of NiTi alloy: powder X-ray diffraction study”. *Materials Science and Engineering A*.

[35] Nygards M (2003). Number of grains necessary to homogenize elastic materials with cubic symmetry. *Mechanics of Materials*.

[36] Sanchez FM, Pulos G, (2008). Use of digital image correlation to determine the mechanical behavior of materials. *Materials Characterization*.

[37] Nam TH, Saburi T, Shimizu KI (1990). Cu-content dependence of shape memory characteristics in Ti-Ni-Cu alloys. *Materials Transactions, JIM*.

[38] Nam TH, Saburi T, Shimizu KI (1990). *Materials Transactions*.

[39] Lagoudlas DC (2008). *Memory Alloys: Moding and Engineering Applications*.

